# The mediating role of depressive symptoms in the relationship between obesity-related triglyceride-glucose index and cognitive status among middle-aged and elderly adults: a large cohort study

**DOI:** 10.3389/fpsyt.2026.1772673

**Published:** 2026-03-03

**Authors:** Yuxuan Jiang, Chaojin Luo, Yuting Zou

**Affiliations:** Department of Rehabilitation Medicine Center, West China Hospital, Sichuan University, Chengdu, Sichuan, China

**Keywords:** cognitive status, depressive symptoms, mediation analysis, obesity index, triglyceride-glucose

## Abstract

**Background:**

Recent research suggests that the obesity-related triglyceride-glucose (TyG) index is associated with cognitive status and depressive symptoms. However, the existing evidence is limited and inconsistent. Furthermore, it remains unclear whether depressive symptoms play a significant role in the relationship between obesity-related TyG indexes and cognitive status.

**Objective:**

This study aims to investigate the relationship among the obesity-related TyG index, cognitive status, and depressive symptoms, as well as the role of depressive symptoms in the association between obesity-related TyG indexes and cognitive status.

**Methods:**

A total of 5,822 participants from the China Health and Retirement Longitudinal Study (CHARLS) were included in this study. Cognitive status was assessed using the Telephone Interview of Cognitive Status (TICS), while depressive symptoms were evaluated using the 10-item version of the Center for Epidemiologic Studies Depression Scale (CES-D-10). TyG and obesity-related indicators (body mass index [BMI], waist circumference [WC], and waist-to-height ratio [WHtR]) were calculated from individual blood and physical examinations. Multivariate linear regression was employed to examine the associations among the obesity-related TyG index, depressive symptoms, and cognitive status. Restricted cubic spline (RCS) analysis was used to test the non-linear relationships between the obesity-related TyG index and both cognitive status and depressive symptoms. Mediation analysis was conducted to assess the role of depressive symptoms in the relationship between the obesity-related TyG index and cognitive status.

**Results:**

The TyG-BMI (coefficient: 0.206; 95% confidence interval [*CI*]: 0.069, 0.343), TyG-WC (0.268; 0.135, 0.402), and TyG-WHtR (0.150; 0.013, 0.286) were positively associated with cognitive status. In contrast, TyG-BMI (-0.147; -0.292, -0.002) and TyG-WC (-0.163; -0.306, -0.021) exhibited a negative relationship with depressive symptoms. RCS analysis indicated a non-linear relationship between TyG-BMI, TyG-WC, and TyG-WHtR and both cognitive status and depressive symptoms. Mediation analysis revealed that depressive symptoms played an important role in the associations between both TyG-BMI and TyG-WC and cognitive status, with mediation proportions of 6.8% and 5.8%, respectively.

**Conclusion:**

Depressive symptoms plays an important role in the relationship between both TyG-BMI and TyG-WC and cognitive status, highlighting depressive symptoms as a potentially relevant pathway that warrants further longitudinal and interventional validation.

## 1 Introduction

As the global population continues to grow and the proportion of older adults steadily increases, dementia has emerged as a significant challenge to public health (1). Dementia is currently the seventh leading cause of death and one of the primary reasons for loss of ability and reliance on others among the elderly worldwide (2). Early cognitive decline often marks the intermediate stage between typical aging and the onset of dementia, which can progress to permanent neurological impairment and significant disability (3). When individuals experience serious cognitive impairment and require daily assistance, it not only affects their quality of life but also imposes a heavy economic and caregiving responsibility on families (4). Currently, there are limited effective treatments for managing dementia (5, 6). Therefore, it is important to prevent the decline of cognitive function.

Depression has become one of the most widespread mental health issues across the world, posing serious challenges to public health (7). Affecting a substantial proportion of the population, it stands as a leading factor contributing to the overall burden of disease worldwide (8). Beyond its psychological symptoms, depression is strongly associated with a range of physical illnesses, such as heart disease, stroke, cancers, disorders of metabolism, and even certain infections (9). The consequences of depression are far-reaching, often diminishing an individual’s daily functioning and social participation, and in severe cases, may result in self-injury or suicidal thoughts (10, 11). Additionally, those living with depression may require considerable support, increasing the demands on caregivers and adding to overall healthcare expenses, which collectively places notable economic and social stress on both families and the broader society (12).

The triglyceride-glucose (TyG) index is a comprehensive measure that integrates fasting triglyceride and glucose levels, serving as an effective alternative biomarker for insulin resistance (IR) (13). The obesity-related TyG index, which incorporates metrics such as body mass index (BMI), waist circumference (WC), and waist-to-height ratio (WHtR), has demonstrated improved sensitivity and specificity in clinical and epidemiological studies (14). Recent research indicates that the obesity-related TyG index is associated with various health outcomes, including cardiovascular disease, gallstones, cognitive function, and depression (15–18). However, evidence regarding the links for cognitive function (17, 19) and depression (18, 20) remains limited and inconsistent. Furthermore, depression may play a crucial role in the relationship between metabolic indicators and cognitive function, presenting new intervention targets for the protection of cognitive function. For instance, depression has been shown to mediate the association between blood urea nitrogen to creatinine ratio and cognitive function (21). However, whether depression plays an important role in the relationship between obesity-related TyG indexes and cognitive function remains unknown.

To address this gap, we performed several analyses in the present study: 1) examining the association between the obesity-related TyG index and cognitive status; 2) evaluating the link between the obesity-related TyG index and depressive symptoms; and 3) assessing whether depressive symptoms plays an important role in the relationship between the obesity-related TyG index and cognitive status. Through this approach, we aim to provide valuable insights that could contribute to strategies for the prevention and management of cognitive impairment and dementia.

## 2 Methods

### 2.1 Study population and design

Participants included in our analysis were from waves 3, 4, and 5 of the China Health and Retirement Longitudinal Study (CHARLS). CHARLS is an ongoing prospective study that has gathered comprehensive data over five survey waves conducted in 2011, 2013, 2015, 2018, and 2020. The initial wave employed a stratified, multistage probability sampling strategy to recruit 17,708 individuals aged 45 years and older, providing a nationally representative sample of middle-aged and elderly adults from 150 districts and counties, as well as 450 communities throughout China. This dataset contains a comprehensive array of information, including demographic backgrounds, health-related behaviors, physical and psychological conditions, as well as laboratory biomarkers. This data was gathered through structured interviews, medical examinations, and blood tests. The study was approved by the Biomedical Ethics Committee of Peking University (approval number: IRB00001052-11015), and written informed consent was obtained from all participants. Additional methodological details are available in previously published article (22). To ensure temporal ordering, obesity-related TyG indexes were assessed at Wave 3 (2015), depressive symptoms at Wave 4 (2018), and cognitive status at Wave 5 (2020).

**Figure 1:**
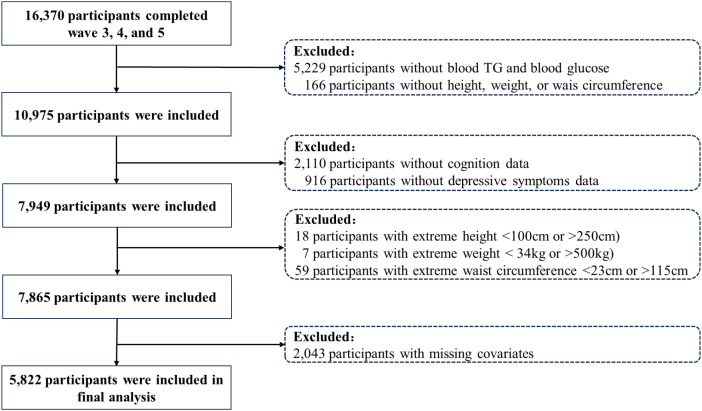
Inclusion and exclusion flowchart.

Among 16,370 individuals who participated in Waves 3–5, we excluded 5,393 participants without available fasting triglycerides, fasting glucose, height, weight, or waist circumference data. We further excluded 2,110 participants without cognitive assessments and 916 without depressive symptom data. In addition, 84 participants with extreme anthropometric values were excluded, as well as 2,043 participants with missing key covariates. The final analytic sample consisted of 5,822 participants. A detailed flowchart of the selection process is presented in **Figure 1**.

### 2.2 Assessment of cognitive status

Global cognitive status was assessed using the CHARLS cognitive battery based on a modified Telephone Interview of Cognitive Status (TICS), which is a brief screening instrument adapted from the Health and Retirement Study (23, 24). This assessment covers two main domains: episodic memory and mental status, capturing both dynamic and acquired aspects of intelligence. Episodic memory was examined by presenting participants with a list of ten Chinese words, which they were asked to recall immediately and again after a short interval. Scores for this domain ranged from 0 to 20, depending on the number of words remembered across both trials. The mental status section included orientation, visuoconstruction, and numerical skills. Orientation was tested by asking participants about the current date, day of the week, month, season, and year, with a maximum score of 5. Visuoconstruction ability was measured by having individuals replicate a given drawing. For numeric ability, participants were tasked with serially subtracting seven from 100 up to five times, with a score range of 0 to 5 based on correct answers. The combined score for mental status (orientation, visuoconstruction, and numeracy) could reach up to 11. Altogether, the total cognitive score spanned from 0 to 31, where higher scores reflected better cognitive status.

### 2.3 Assessment of depressive symptoms

Depressive symptoms were measured using the 10-item version of the Center for Epidemiologic Studies Depression Scale (CES-D-10), a tool widely recognized for its reliability in screening for depressive symptoms among Chinese adults in midlife and older age (25). Participants responded to questions about how often they experienced various negative emotions over the past week, with answer choices scored from 0 (rarely or never) to 3 (most of the time). The sum of these responses produced a total score between 0 and 30, where higher values reflected more severe depressive symptoms. As the CES-D-10 reflects depressive symptoms during the past week, it represents short-term symptom burden rather than chronic depressive symptoms.

### 2.4 Assessment of obesity-related TyG index

After participants completed face-to-face interviews in their local communities, fasting blood samples (8 ml) were collected at nearby hospitals or Centers for Disease Control by trained medical staff. Part of the sample was analyzed on site for basic hematological parameters within two hours, such as hemoglobin, hematocrit, white blood cell and platelet counts, and mean corpuscular volume. The remaining blood was promptly frozen at -20 °C and shipped to the central laboratory in Beijing for further biochemical assays, including fasting plasma glucose and triglyceride measurements. Height and weight were measured by trained staff using a stadiometer and a calibrated scale with participants barefoot and in light clothing. Waist circumference was measured at the level of the navel in a standing position using a flexible tape held horizontal and snug but not tight.

Using these values, the TyG index was calculated as ln[fasting triglyceride (mg/dL) × fasting glucose (mg/dL)/2]. To capture the impact of both metabolic and anthropometric factors, three composites indexes were constructed: TyG-BMI (TyG index multiplied by body mass index), TyG-WC (TyG index multiplied by waist circumference), and TyG-WHtR (TyG index multiplied by waist-to-height ratio). BMI was calculated as weight (kg)/height (m)^2^, waist circumference (WC) was measured in centimeters, and WHtR was calculated as WC (cm)/height (cm). For all subsequent statistical analyses, each of these indexes was standardized by dividing by its standard deviation. More comprehensive details on blood sample collection and laboratory methods are documented in prior research (26).

### 2.5 Covariates

In line with established literature (17–20), we included a comprehensive set of potential confounders encompassing sociodemographic, lifestyle, and health-related factors. Sociodemographic covariates included sex (male and female), age, residence (urban and rural), marital status (live with partner and living without partner), and education. Education was categorized into three levels, including elementary education (no formal education, did not complete primary school, home schooling, or elementary school), secondary education (middle school, high school, or vocational school), and higher education (associate degree, bachelor’s degree, master’s degree, or doctoral degree). Lifestyle covariates included smoking status and alcohol consumption. Health-related covariates included hypertension, diabetes, and dyslipidemia, defined as the respondent’s answer to the question regarding whether or not a doctor has told the respondent they had a specific condition.

### 2.6 Statistical analysis

Participants were sorted into four groups according to the quartile distribution (Q1–Q4) of each cognition, determined by the 25th, 50th, and 75th percentiles. For continuous data, results are given as mean values with their standard deviations (SD), and categorical variables are presented as counts and percentages within each subgroup. Comparative analyses across these quartiles were performed using analysis of variance and chi-squared tests.

We employed a multivariate linear regression modeling strategy to examine the relationships between obesity-related TyG indexes and both cognitive status and depressive symptoms. Model 1 did not control for any confounding variables. Model 2 accounted for sex, age, education, and marital status, as well as residence. Model 3 further adjusted for alcohol consumption and smoking. Model 4 additionally adjusted for hypertension, diabetes, and dyslipidemia. The estimated coefficients along with their corresponding 95% confidence intervals (CI) were reported.

We utilized restricted cubic spline (RCS) regression to investigate potential non-linear relationships between TyG indexes and both cognitive status and depressive symptoms, while adjusting for the same covariates as in Model 4 of the multivariate linear regression.

To investigate possible mechanistic pathways, mediation analyses were conducted based on a set of fundamental assumptions, including the absence of unmeasured confounding among exposure, mediator, and outcome (27). The mediation framework quantified the total effect (TE), natural direct effect (NDE), and natural indirect effect (NIE) as defined in the natural effect model methodology (28). Briefly, TE captures the overall association between the exposure and the outcome. NDE reflects the effect of the exposure on the outcome not operating through the mediator, with the mediator fixed at the level it would naturally take under the unexposed condition. NIE reflects the effect operating through the mediator, representing the portion of the association attributable to exposure-induced changes in the mediator. The proportion of the mediated (PM) was calculated as the ratio of NIE to TE. Mediation analysis utilized the “mediation” package in R, with confidence intervals assessed via 2,000 bootstrap iterations (29).

The reliability of the results was further examined through a series of sensitivity analyses. First, we additionally adjusted for medication use in the fully adjusted multivariable models to evaluate potential confounding by pharmacological treatment. Second, individuals with missing covariate data were retained in the analysis by applying multiple imputation techniques. Third, we extended the analysis to include participants with extreme values for height, weight, and waist circumference. Finally, we conducted additional analyses focusing on distinct cognitive domains, such as episodic memory and mental status.

All statistical computations were conducted using R (version 4.4.0). Statistical significance was defined by two-tailed p-values less than 0.05.

## 3 Results

### 3.1 Baseline characteristics

**Table 1** presented the baseline characteristics of participants included in our study stratified by quartiles of cognitive status. The average age of participants was 59.04 ± 8.15 years, with a female representation of 52.4%. Notably, mean age decreased across increasing cognitive status quartiles: participants in Q1 had a mean age of 61.32 ± 8.25 years, whereas those in Q4 had a mean age of 56.79 ± 7.87 years. A higher percentage of females were found in Q1 (63.3%), whereas males were predominant in Q4 (52.6%). Most participants (86.3%) lived with their partners, and a significant majority resided in rural areas (79.9%). Detailed information about other characteristics were provided in **Table 1**.

**Table 1:** Baseline characteristics of study participants by cognitive status quartiles [n (%) or Mean ± SD].

Characteristic	Overall N = 5,822^1^	Q1 N = 1,486^1^	Q2 N = 1,455^1^	Q3 N = 1,479^1^	Q4 N = 1,402^1^	p-value^2^
Age	59.04 ± 8.15	61.32 ± 8.25	59.36 ± 7.91	58.56 ± 7.90	56.79 ± 7.87	<0.001
Gender						<0.001
Female	3,048 (52.4%)	940 (63.3%)	769 (52.9%)	675 (45.6%)	664 (47.4%)	
Male	2,774 (47.6%)	546 (36.7%)	686 (47.1%)	804 (54.4%)	738 (52.6%)	
Marriage						<0.001
Live with partner	5,024 (86.3%)	1,215 (81.8%)	1,241 (85.3%)	1,316 (89.0%)	1,252 (89.3%)	
Live without partner	798 (13.7%)	271 (18.2%)	214 (14.7%)	163 (11.0%)	150 (10.7%)	
Education						<0.001
Elementary education	3,538 (60.8%)	1,298 (87.3%)	1,002 (68.9%)	755 (51.0%)	483 (34.5%)	
Secondary education	2,191 (37.6%)	183 (12.3%)	444 (30.5%)	694 (46.9%)	870 (62.1%)	
Higher education	93 (1.6%)	5 (0.3%)	9 (0.6%)	30 (2.0%)	49 (3.5%)	
Residence						<0.001
Urban	1,172 (20.1%)	137 (9.2%)	240 (16.5%)	334 (22.6%)	461 (32.9%)	
Rural	4,650 (79.9%)	1,349 (90.8%)	1,215 (83.5%)	1,145 (77.4%)	941 (67.1%)	
Alcohol						<0.001
No	3,065 (52.6%)	871 (58.6%)	770 (52.9%)	739 (50.0%)	685 (48.9%)	
Yes	2,757 (47.4%)	615 (41.4%)	685 (47.1%)	740 (50.0%)	717 (51.1%)	
Smoke						<0.001
No	3,279 (56.3%)	927 (62.4%)	813 (55.9%)	767 (51.9%)	772 (55.1%)	
Yes	2,543 (43.7%)	559 (37.6%)	642 (44.1%)	712 (48.1%)	630 (44.9%)	
Hypertension						<0.001
No	4,000 (68.7%)	969 (65.2%)	979 (67.3%)	1,036 (70.0%)	1,016 (72.5%)	
Yes	1,822 (31.3%)	517 (34.8%)	476 (32.7%)	443 (30.0%)	386 (27.5%)	
Diabetes						0.215
No	5,251 (90.2%)	1,327 (89.3%)	1,304 (89.6%)	1,338 (90.5%)	1,282 (91.4%)	
Yes	571 (9.8%)	159 (10.7%)	151 (10.4%)	141 (9.5%)	120 (8.6%)	
Dyslipidemia						0.004
No	4,688 (80.5%)	1,234 (83.0%)	1,173 (80.6%)	1,192 (80.6%)	1,089 (77.7%)	
Yes	1,134 (19.5%)	252 (17.0%)	282 (19.4%)	287 (19.4%)	313 (22.3%)	
TyG-BMI	211.36 ± 40.85	208.02 ± 41.31	210.85 ± 38.56	212.02 ± 41.42	214.73 ± 41.82	<0.001
TyG-WC	752.19 ± 132.45	736.38 ± 138.99	750.57 ± 134.83	758.01 ± 129.38	764.48 ± 124.24	<0.001
TyG-WHtR	4.74 ± 0.84	4.73 ± 0.91	4.74 ± 0.85	4.75 ± 0.81	4.76 ± 0.78	0.868

a *P* value was calculated for continuous variables by t-test and categorical variables by Pearson’s Chi-squared test.

SD, standard deviation; TyG, triglyceride-glucose; BMI, body mass index; WC, waist circumference; WHtR, waist-to-height ratio.

### 3.2 Associations among obesity-related TyG indexes, cognitive status and depressive symptoms

**Figure 2:**
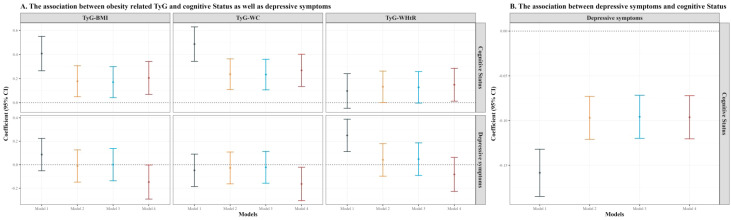
Part **(A)**: The association between obesity-related TyG indexes (TyG-BMI, TyG-WC, TyG-WHtR) and cognitive status as well as depressive symptoms. Part **(B)**: The association between depressive symptoms and cognitive status. Model 1 is adjusted for no covariates, Model 2 adjusts for sex, age, education, marital status, and residence, Model 3 further adjusts for alcohol consumption and smoking, and Model 4 includes adjustments for hypertension, diabetes, and dyslipidemia.TyG, triglyceride-glucose; BMI, body mass index; WC, waist circumference; WHtR, waist-to-height ratio. CI, confidence interval.

**Figure 2** illustrated the associations among obesity-related TyG indexes (TyG-BMI, TyG-WC, and TyG-WHtR), depressive symptoms, and cognitive status. Panel A shows that TyG-BMI, TyG-WC, and TyG-WHtR were positively associated with cognitive status (upper row), while TyG-WC and TyG-WHtR exhibited a negative relationship with depressive symptoms (lower row) after full adjustment for covariates. Specifically, the coefficients (95% CI) for TyG-BMI, TyG-WC, and TyG-WHtR in relation to cognitive status were 0.206 (0.069 ~ 0.343), 0.268 (0.135 ~ 0.402), and 0.150 (0.013 ~ 0.286), respectively, in Model 4. The coefficients (95% CI) for TyG-BMI and TyG-WC in relation to depressive symptoms were -0.147 (-0.292 ~ -0.002) and -0.163 (-0.306 ~ -0.021), respectively, in Model 4. Further details were provided in **Supplementary Table S1**. Panel B demonstrates the relationship between depressive symptoms and cognitive status. Across all models, higher depressive symptoms scores were significantly associated with poorer cognitive status. In Model 4, the coefficient (95% CI) was -1.096 (-1.371 ~ -0.820). Additional information can be found in **Supplementary Table S2**.

### 3.3 Non-linear relationships between obesity-related TyG and both cognitive status and depressive symptoms

**Figure 3:**
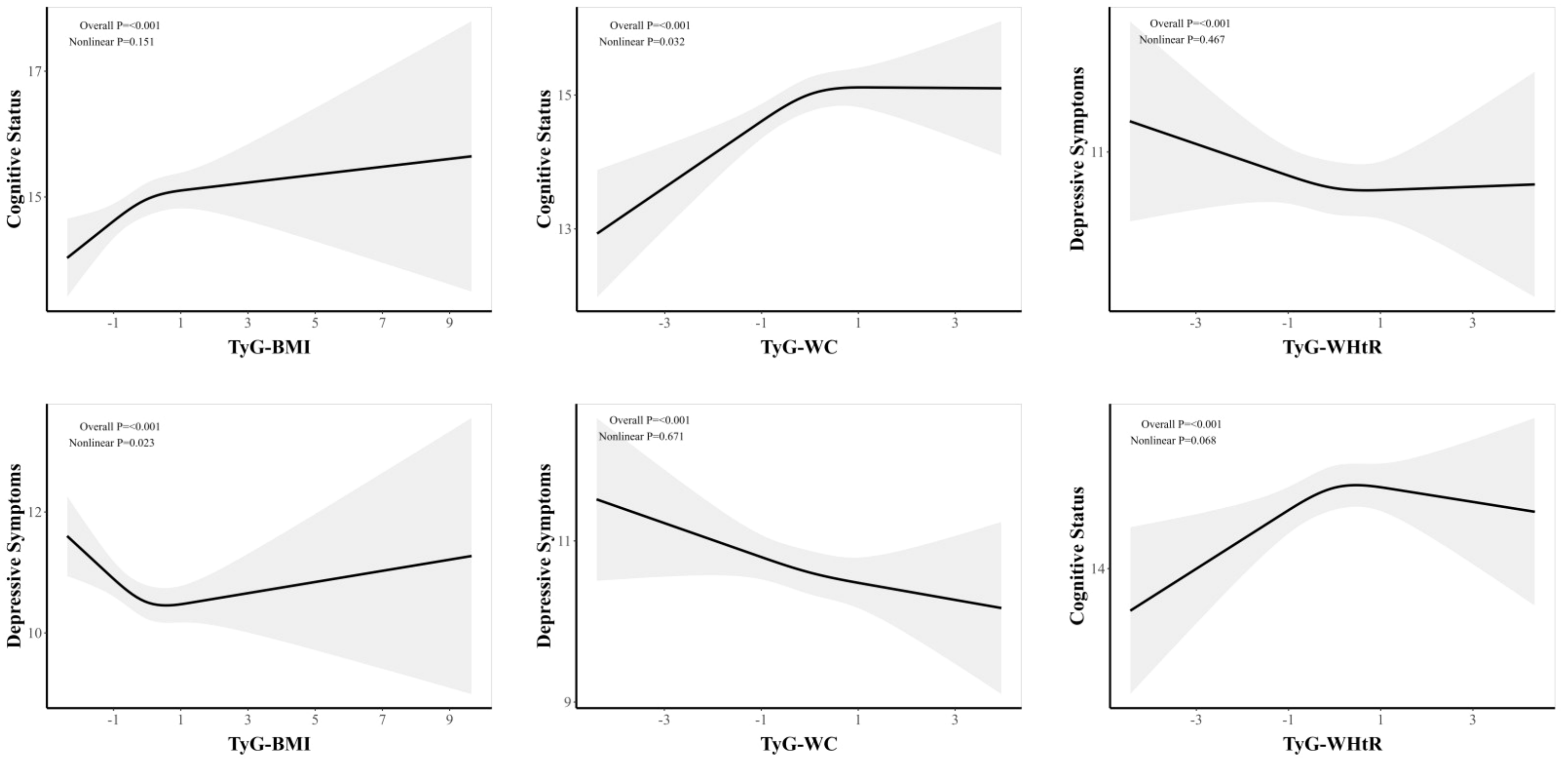
Non-linear relationships between obesity-related TyG and both cognitive status and depressive symptoms. TyG, triglyceride-glucose; BMI, body mass index; WC, waist circumference; WHtR, waist-to-height ratio. CI, confidence interval. Model adjusted for sex, age, education, and marital status, residence, alcohol consumption, smoking, hypertension, diabetes, and dyslipidemia.

**Figure 3** illustrated the non-linear relationships among three obesity-related TyG indexes (TyG-BMI, TyG-WC, and TyG-WHtR) in relation to cognitive status and depressive symptoms. For instances, the analysis revealed a significant positive association between TyG-WC and cognitive status, with an overall p-value of <0.001 and a significant nonlinear p-value of 0.032. In contrast, a significant negative association was observed between TyG-WC and depressive symptoms, indicating that higher TyG-WC values are associated with lower depressive symptoms. The overall p-value for this relationship is also <0.001, while the nonlinear p-value of 0.671. **Figure 3** provided other relationships between obesity-related TyG indexes and both cognitive status and depressive symptoms.

### 3.4 Mediation analysis

**Figure 4:**
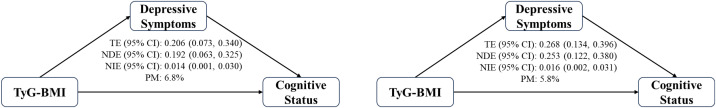
Mediation analysis of depressive symptoms in the association between obesity-related TyG indexes and cognitive status. TyG, triglyceride-glucose; BMI, body mass index; WC, waist circumference; WHtR, waist-to-height ratio. CI, confidence interval; TE, total effect; NDE, natural direct effect; NIE, natural indirect effect; PM, proportion of mediated. Model adjusted for sex, age, education, and marital status, residence, alcohol consumption, smoking, hypertension, diabetes, and dyslipidemia.

Mediation analyses were conducted to investigate whether depressive symptoms play an important role in the relationship between obesity-related TyG indexes and cognitive status. **Figure 4** illustrated that depressive symptoms played an important role in the association between both TyG-BMI and TyG-WC with cognitive status. Specifically, the NIEs of TyG-BMI and TyG-WC on cognitive status via depressive symptoms were 0.014 (95% CI: 0.001 ~ 0.030; p = 0.038) and 0.016 (95% CI: 0.002 ~ 0.031; p = 0.025), respectively. Furthermore, the proportion mediated was 6.8% (PM = 0.068; 95% CI: 0.003 ~ 0.195; p = 0.039) for TyG-BMI and 5.8% (PM = 0.058; 95% CI: 0.007 ~ 0.138; p = 0.025) for TyG-WC. The numeric results corresponding to **Figure 4** were shown in **Supplementary Table S3**.

### 3.5 Sensitivity analysis

Our sensitivity analyses confirmed the robustness of all findings. After further adjusting for medication use, the positive associations between TyG-BMI, TyG-WC, and TyG-WHtR and cognitive status remained significant and of similar magnitude (**Supplementary Table S4**). Even after multiple imputation for missing data, depressive symptoms remained play an important role in the relationship between both TyG-BMI and TyG-WC and cognitive status (**Supplementary Table S5**). Furthermore, after accounting for extreme values of BMI, WC, and WHtR, depressive symptoms continued to play an important role in the association between TyG-WC and cognitive status (**Supplementary Table S6**). Additionally, depressive symptoms also played an important role in the relationships between TyG-BMI and TyG-WC and distinct cognitive domains, including episodic memory and mental status (**Supplementary Tables S7, 8**). These findings support the robustness of the observed associations and suggest that depressive symptom burden may be a relevant correlate in the TyG–cognition relationship, warranting further longitudinal and interventional validation.

## 4 Discussion

In a large nationwide cohort study, we observed positive associations between TyG-BMI, TyG-WC, and TyG-WHtR and cognitive status. Conversely, TyG-BMI and TyG-WC were found to be negatively correlated with depressive symptoms. Additionally, our study is the largest epidemiological investigation to reveal that depressive symptoms serve as a significant mediator in the relationship between both TyG-BMI and TyG-WC and cognitive status.

### 4.1 Comparison with previous studies

Previous research has largely suggested that higher insulin resistance and obesity metrics correlate with worse cognitive outcomes, including faster cognitive decline (30). In contrast, our study found a positive or even inverted U-shaped association between obesity-related TyG indices (TyG-BMI and TyG-WC) and cognitive function. Notably, few studies have specifically examined TyG-BMI or TyG-WC in this context, and the available findings have been inconsistent. While most literature links elevated TyG index or combined metrics with cognitive impairment (30, 31), our observations indicate that moderate increases in these indices were associated with better cognitive performance. This discrepancy highlights the need for careful interpretation and further investigation.

In contrast, evidence specifically focusing on obesity related TyG indices is still limited and mixed, which makes direct comparison challenging. For instance, a cohort study of older Chinese adults (n = 1,125) reported that higher TyG-WC was associated with lower odds of cognitive impairment and better Mini-Mental State Examination score (17). Similarly, another study of 855 non-demented individuals found that those with elevated TyG-BMI had a decreased risk of cognitive decline (19). However, other studies report the opposite. For example, a longitudinal analysis of non-diabetic adults found that higher TyG-BMI was associated with higher risk of incident mild cognitive impairment over follow up (32). Taken together, the literature suggests that the association between obesity related TyG indices and cognition is not yet settled and may differ by population and analytic approach.

One important explanation is reverse causality in later life, because preclinical cognitive decline and impending dementia can be accompanied by changes in appetite, diet, physical activity, and body composition. Weight and central adiposity may decline years before a dementia diagnosis, which can make higher adiposity-based indices appear neutral or even beneficial in observational analyses with shorter follow up or older samples (33–35). In addition, the reported inverted U-shaped pattern should be interpreted cautiously, because selective attrition and selective survival can lead to underrepresentation of individuals with severe metabolic dysregulation and poorer cognition at the upper end of TyG BMI or TyG WC. This form of selection can distort dose response relationships and can produce an apparent downturn in spline analyses even when the underlying association is monotonic (36–38). Finally, sparse data at exposure extremes and model flexibility can further amplify instability in estimated curves. Similar nonlinear patterns have been reported for TyG WC and cognitive impairment in other Chinese samples, supporting the need to view the inverted U-shaped association as exploratory and to prioritize replication in studies with repeated metabolic measures and approaches that explicitly address attrition (17).

### 4.2 Potential mechanisms

Obesity-related TyG indices (TyG-BMI, TyG-WC) capture the combined influence of adiposity and insulin resistance, which may help explain their associations with cognitive status. Brain insulin resistance has been increasingly recognized as a contributor to cognitive aging through disrupted neuronal insulin signaling, impaired synaptic plasticity, and altered proteostasis (39). The beneficial associations observed within a specific range may be explained by metabolic and vascular pathways influenced by insulin resistance and obesity (40). In particular, insulin resistance is linked to endothelial dysfunction and cerebral small vessel disease, and TyG-related measures have also been associated with small vessel disease burden and cognitive impairment in prior studies (41). Within this range, elevated levels of TyG-BMI, TyG-WC, and TyG-WHtR may indicate a balance in energy metabolism, potentially safeguarding cognitive status by enhancing insulin sensitivity and glucose utilization in the brain (42). Human neuroimaging evidence suggests that higher peripheral insulin resistance is associated with reduced cerebral glucose metabolism in vulnerable groups, supporting the relevance of brain glucose utilization for cognition (43). Conversely, both excessively high and low levels of these indexes may reflect metabolic imbalance, including hyperinsulinemia or inadequate energy supply to the brain, which may be detrimental to cognitive health (44). At the high end, obesity and insulin resistance may promote oxidative stress and neuroinflammation and may impair blood–brain barrier integrity, which can precede and accelerate cognitive decline (45). These mechanisms could explain the U-shaped relationship noted in our study.

With respect to depressive symptoms, metabolic dysregulation and depressive symptoms share overlapping biological pathways, particularly chronic low-grade inflammation, which can influence mood-related neural circuits (46, 46). At lower concentrations, higher TyG-BMI and TyG-WHtR were associated with lower depressive symptom scores, potentially reflecting improved metabolic homeostasis (47). In contrast, at higher concentrations, further increases were associated with greater depressive symptoms, possibly through activation of stress-related pathways, including hypothalamic-pituitary-adrenal (HPA) axis dysregulation (48). HPA-axis hyperactivity is a frequently reported neuroendocrine feature in major depressive symptom and may contribute to hippocampal vulnerability and affective symptoms (49, 50). These findings reiterates the importance of maintaining metabolic and obesity indicators within appropriate ranges. Furthermore, hypoglycemia is a potent physiological stressor that triggers autonomic and neuroendocrine counterregulation and can produce anxiety-like symptoms, which may contribute to depressive symptomatology in susceptible individuals (51–53). In addition, because TyG-BMI and TyG-WHtR incorporate fasting triglycerides, fasting glucose, and adiposity, very low levels may be accompanied by reduced dopaminergic neuronal activity, which has been linked to depressive symptoms (54). Consistent with this, studies indicate that dopaminergic systems and reward processing are implicated in depressive phenotypes, and experimental work shows that dopaminergic neuronal activity can be sensitive to glucose availability (55–57).

### 4.3 Mediation analysis

Additionally, to our knowledge, our study represents the first epidemiological investigation to demonstrate that depressive symptoms plays an important role in the relationship between both TyG-BMI and TyG-WC and cognitive status. This pivotal finding highlights the intricate interplay between metabolic indicators and mental health in the context of cognitive decline. Specifically, we found that depressive symptoms accounted for a mediation proportion of 6.8% with respect to TyG-BMI and 5.8% in relation to TyG-WC. These findings indicate that depressive symptom burden may be one pathway linking metabolic risk profiles and cognitive performance. However, given the observational design and the assumptions required for mediation, these results should be interpreted as indirect associations rather than definitive causal mechanisms. Future longitudinal studies and intervention trials are warranted to determine whether reducing depressive symptoms influences cognitive outcomes.

### 4.4 Strengths and limitations

Our research demonstrates several significant strengths. First and foremost, we utilized a large national population cohort, which enabled us to collect a comprehensive set of variables that encompass demographic characteristics, behavioral patterns, health status, as well as physiological and biochemical indicators. This extensive data collection enhances the reliability and precision of our findings. Secondly, the timing of our assessments for obesity-related TyG indexes, depressive symptoms, and cognitive status, conducted in 2015, 2018, and 2020, respectively, minimizes the risk of causal inversion, thereby strengthening the validity of our conclusions about the temporal relationships between these variables. Thirdly, our study is novel in that it is the first to explicitly identify and analyze the mediating role of depressive symptoms in the relationship between obesity-related TyG indexes and cognitive status.

Several limitations should be acknowledged. First, although we established temporal ordering (2015→2018→2020), this observational design remains susceptible to residual and unmeasured confounding, and causal interpretations should be made cautiously. Second, selection and attrition across waves may introduce bias, as the analytic sample required complete participation and key measurements. Third, depressive symptoms were assessed using the CES-D-10, which captures experiences over the preceding week. Consequently, our mediation analysis reflects short-term depressive symptom burden rather than chronic or recurrent depression. This mismatch in time scale may result in measurement misclassification and further limits causal interpretation. Fourth, cognitive performance was measured using the modified TICS-based battery in CHARLS, which primarily serves as a screening tool for global cognitive status and does not provide fine-grained assessment of specific cognitive functions.

Future studies should incorporate repeated measurements of metabolic and anthropometric indicators to characterize trajectories, and evaluate incident mild cognitive impairment as clinical endpoints. Applying alternative causal inference approaches (e.g., marginal structural models or instrumental variable methods where feasible) may further strengthen causal interpretation. Interventional research targeting depressive symptoms among individuals with adverse metabolic profiles may also help clarify whether improving mental health can mitigate cognitive decline.

## 5 Conclusion

In conclusion, this extensive nationwide cohort study in China provides compelling evidence of the positive associations between TyG-BMI, TyG-WC, and TyG-WHtR with cognitive status. Furthermore, our findings reveal a noteworthy negative correlation between TyG-BMI and TyG-WC with depressive symptoms, underscoring the potential protective effects of these indexes on mental health. Importantly, our study is the largest epidemiological investigation to demonstrate that depressive symptoms play an important role in the relationship between TyG-BMI and TyG-WC and cognitive status. These insights not only enhance our understanding of the complex interplay between metabolic indexes and mental health but also suggest critical avenues for future research aimed at developing targeted interventions to mitigate cognitive decline and improve overall mental well-being.

## Data Availability

The data used in this paper is available in public and can be accessed at China Health and Retirement Longitudinal Study (CHARLS) http://charls.pku.edu.cn/.
