# Supplementary material for: The mediating role of depressive symptoms in the relationship between obesity-related triglyceride-glucose index and cognitive status among middle-aged and elderly adults: a large cohort study
Source: Front Psychiatry. 2026 Mar 3;17:1772673. doi: 10.3389/fpsyt.2026.1772673 (PMC12993277; doi:10.3389/fpsyt.2026.1772673)
Supplement: Supplementary file 1 [file Table1.docx]

**Supplemental Materials**

**The mediating role of depressive symptoms in the relationship between obesity-related triglyceride glucose index and cognitive status among middle-aged and elderly adults: A large cohort study**

**Contents**

[Table S1 The relationships between obesity-related TyG index and both cognitive status and depressive symptoms 3](#_Toc219158319)

[Table S2 The relationships between depressive symptoms and cognitive status 5](#_Toc219158320)

[Table S3 Mediation analyses of the mediating role of depressive symptoms in the relationship between obesity-related TyG index and cognitive status 6](#_Toc219158321)

[Table S1 Sensitivity analyses of the relationships between obesity-related TyG index and both cognitive status after further adjusting medication 7](#_Toc219158322)

[Table S4 Sensitivity analyses of the mediating role of depressive symptoms in the relationship between obesity-related TyG index and cognitive status 8](#_Toc219158323)

[Table S5 Sensitivity analyses of the mediating role of depressive symptoms in the relationship between obesity-related TyG index and cognitive status after including extreme values 9](#_Toc219158324)

[Table S6 Sensitivity analyses of the mediating role of depressive symptoms in the relationship between obesity-related TyG index and episodic memory 10](#_Toc219158325)

[Table S7 Sensitivity analyses of the mediating role of depressive symptoms in the relationship between obesity-related TyG index and mental status 11](#_Toc219158326)

**Table S1 The relationships between obesity-related TyG index and both cognitive status and depressive symptoms**

| **Outcome** | **Exposure** | **Model** | **Coefficient (95% *CI*)** | ***P*** |
| --- | --- | --- | --- | --- |
| Cognitive status | TyG-BMI | Model 1 | 0.408(0.265,0.552) | <0.001 |
| Cognitive status | TyG-BMI | Model 2 | 0.179(0.050,0.307) | 0.007 |
| Cognitive status | TyG-BMI | Model 3 | 0.171(0.042,0.300) | 0.009 |
| Cognitive status | TyG-BMI | Model 4 | 0.206(0.069,0.343) | 0.003 |
| Cognitive status | TyG-WC | Model 1 | 0.487(0.344,0.630) | <0.001 |
| Cognitive status | TyG-WC | Model 2 | 0.237(0.110,0.364) | <0.001 |
| Cognitive status | TyG-WC | Model 3 | 0.234(0.107,0.361) | <0.001 |
| Cognitive status | TyG-WC | Model 4 | 0.268(0.135,0.402) | <0.001 |
| Cognitive status | TyG-WHtR | Model 1 | 0.097(-0.046,0.241) | 0.185 |
| Cognitive status | TyG-WHtR | Model 2 | 0.132(0.001,0.262) | 0.048 |
| Cognitive status | TyG-WHtR | Model 3 | 0.128(-0.003,0.258) | 0.055 |
| Cognitive status | TyG-WHtR | Model 4 | 0.150(0.013,0.286) | 0.032 |
| Depressive symptoms | TyG-BMI | Model 1 | 0.087(-0.051,0.225) | 0.218 |
| Depressive symptoms | TyG-BMI | Model 2 | -0.010(-0.148,0.127) | 0.881 |
| Depressive symptoms | TyG-BMI | Model 3 | 0.001(-0.137,0.138) | 0.992 |
| Depressive symptoms | TyG-BMI | Model 4 | -0.147(-0.292,-0.002) | 0.046 |
| Depressive symptoms | TyG-WC | Model 1 | -0.048(-0.186,0.090) | 0.498 |
| Depressive symptoms | TyG-WC | Model 2 | -0.027(-0.163,0.109) | 0.697 |
| Depressive symptoms | TyG-WC | Model 3 | -0.022(-0.157,0.114) | 0.752 |
| Depressive symptoms | TyG-WC | Model 4 | -0.163(-0.306,-0.021) | 0.024 |
| Depressive symptoms | TyG-WHtR | Model 1 | 0.250(0.112,0.388) | <0.001 |
| Depressive symptoms | TyG-WHtR | Model 2 | 0.041(-0.098,0.180) | 0.559 |
| Depressive symptoms | TyG-WHtR | Model 3 | 0.048(-0.091,0.187) | 0.500 |
| Depressive symptoms | TyG-WHtR | Model 4 | -0.082(-0.227,0.063) | 0.268 |

Abbreviations: TyG: triglyceride-glucose; BMI: body mass index; WC: waist circumference; WHtR: waist-to-height ratio. CI, confidence interval.

Model 1 adjusted for no covariates.

Model 2 adjusted for sex, age, education, and marital status, and residence.

Model 3 adjusted for sex, age, education, and marital status, residence, alcohol consumption, and smoking.

Model 4 adjusted for sex, age, education, and marital status, residence, alcohol consumption, smoking, hypertension, diabetes, and dyslipidemia.

**Table S2 The relationships between depressive symptoms and cognitive status**

| **Outcome** | **Exposure** | **Model** | **Coefficient (95% *CI*)** | ***P*** |
| --- | --- | --- | --- | --- |
| Cognitive status | Depressive symptoms | Model 1 | -0.162(-0.188,-0.135) | <0.001 |
| Cognitive status | Depressive symptoms | Model 2 | -0.097(-0.122,-0.073) | <0.001 |
| Cognitive status | Depressive symptoms | Model 3 | -0.096(-0.120,-0.071) | <0.001 |
| Cognitive status | Depressive symptoms | Model 4 | -0.094(-0.118,-0.070) | <0.001 |

Abbreviations: CI, confidence interval.

Model 1 adjusted for no covariates.

Model 2 adjusted for sex, age, education, and marital status, and residence.

Model 3 adjusted for sex, age, education, and marital status, residence, alcohol consumption, and smoking.

Model 4 adjusted for sex, age, education, and marital status, residence, alcohol consumption, smoking, hypertension, diabetes, and dyslipidemia.

**Table S3 Mediation analyses of the mediating role of depressive symptoms in the relationship between obesity-related TyG index and cognitive status**

| **Exposure** | **Effect** | **Coefficient (95% *CI*)** | ***P*** |
| --- | --- | --- | --- |
| TyG-BMI | NIE | 0.014(0.001,0.030) | 0.038 |
| TyG-BMI | NDE | 0.192(0.063,0.325) | 0.001 |
| TyG-BMI | TE | 0.206(0.073,0.340) | 0.001 |
| TyG-BMI | PM | 0.068(0.003,0.195) | 0.039 |
| TyG-WC | NIE | 0.016(0.002,0.031) | 0.025 |
| TyG-WC | NDE | 0.253(0.122,0.380) | <0.001 |
| TyG-WC | TE | 0.268(0.134,0.396) | <0.001 |
| TyG-WC | PM | 0.058(0.007,0.138) | 0.025 |
| TyG-WHtR | NIE | 0.008(-0.006,0.022) | 0.268 |
| TyG-WHtR | NDE | 0.142(0.001,0.275) | 0.048 |
| TyG-WHtR | TE | 0.150(0.009,0.284) | 0.036 |
| TyG-WHtR | PM | 0.053(-0.076,0.357) | 0.282 |

Abbreviations: TyG: triglyceride-glucose; BMI: body mass index; WC: waist circumference; WHtR: waist-to-height ratio. CI, confidence interval; TE: total effect NDE: natural direct effect; NIE: natural indirect effect.

Model adjusted for sex, age, education, and marital status, residence, alcohol consumption, smoking, hypertension, diabetes, and dyslipidemia.

**Table S4 Sensitivity analyses of the relationships between obesity-related TyG index and both cognitive status after further adjusting medication**

| **Outcome** | **Exposure** | **Coefficient (95% *CI*)** | ***P*** |
| --- | --- | --- | --- |
| Cognitive status | TyG-BMI | 0.215(0.073,0.357) | 0.003 |
| Cognitive status | TyG-WC | 0.268(0.130,0.407) | <0.001 |
| Cognitive status | TyG-WHtR | 0.151(0.010,0.293) | 0.036 |

Abbreviations: TyG: triglyceride-glucose; BMI: body mass index; WC: waist circumference; WHtR: waist-to-height ratio. CI, confidence interval

Model adjusted for sex, age, education, and marital status, residence, alcohol consumption, smoking, hypertension, diabetes, dyslipidemia and medication.

**Table S5 Sensitivity analyses of the mediating role of depressive symptoms in the relationship between obesity-related TyG index and cognitive status**

| **Exposure** | **Effect** | **Coefficient (95% *CI*)** | ***P*** |
| --- | --- | --- | --- |
| TyG-BMI | NIE | 0.014(0.001,0.029) | 0.035 |
| TyG-BMI | NDE | 0.192(0.059,0.324) | 0.002 |
| TyG-BMI | TE | 0.206(0.07,0.339) | 0.002 |
| TyG-BMI | PM | 0.068(0.004,0.211) | 0.037 |
| TyG-WC | NIE | 0.016(0.001,0.031) | 0.027 |
| TyG-WC | NDE | 0.255(0.13,0.388) | <0.001 |
| TyG-WC | TE | 0.27(0.144,0.404) | <0.001 |
| TyG-WC | PM | 0.058(0.007,0.139) | 0.027 |
| TyG-WHtR | NIE | 0.008(-0.006,0.023) | 0.278 |
| TyG-WHtR | NDE | 0.142(0.007,0.283) | 0.040 |
| TyG-WHtR | TE | 0.15(0.012,0.294) | 0.038 |
| TyG-WHtR | PM | 0.053(-0.1,0.281) | 0.300 |

Abbreviations: TyG: triglyceride-glucose; BMI: body mass index; WC: waist circumference; WHtR: waist-to-height ratio. CI, confidence interval; TE: total effect NDE: natural direct effect; NIE: natural indirect effect.

Model adjusted for sex, age, education, and marital status, residence, alcohol consumption, smoking, hypertension, diabetes, and dyslipidemia.

**Table S6 Sensitivity analyses of the mediating role of depressive symptoms in the relationship between obesity-related TyG index and cognitive status after including extreme values**

| **Exposure** | **Effect** | **Coefficient (95% *CI*)** | ***P*** |
| --- | --- | --- | --- |
| TyG-BMI | NIE | 0.006(0.004,2.451) | 0.011 |
| TyG-BMI | NDE | -0.084(-11.163,22.624) | 0.534 |
| TyG-BMI | TE | -0.078(-9.925,24.797) | 0.567 |
| TyG-BMI | PM | -0.073(-0.493,0.711) | 0.564 |
| TyG-WC | NIE | 0.018(0.007,0.036) | 0.007 |
| TyG-WC | NDE | 0.226(0.118,0.42) | <0.001 |
| TyG-WC | TE | 0.244(0.136,0.445) | <0.001 |
| TyG-WC | PM | 0.073(0.025,0.155) | 0.007 |
| TyG-WHtR | NIE | 0.007(0.004,0.117) | 0.033 |
| TyG-WHtR | NDE | -0.06(-0.084,1.28) | 0.717 |
| TyG-WHtR | TE | -0.053(-0.077,1.36) | 0.722 |
| TyG-WHtR | PM | -0.14(-0.24,0.23) | 0.689 |

Abbreviations: TyG: triglyceride-glucose; BMI: body mass index; WC: waist circumference; WHtR: waist-to-height ratio. CI, confidence interval; TE: total effect NDE: natural direct effect; NIE: natural indirect effect.

Model adjusted for sex, age, education, and marital status, residence, alcohol consumption, smoking, hypertension, diabetes, and dyslipidemia.

**Table S7 Sensitivity analyses of the mediating role of depressive symptoms in the relationship between obesity-related TyG index and episodic memory**

| **Exposure** | **Effect** | **Coefficient (95% *CI*)** | ***P*** |
| --- | --- | --- | --- |
| TyG-BMI | NIE | 0.009(0,0.02) | 0.038 |
| TyG-BMI | NDE | 0.121(0.009,0.224) | 0.032 |
| TyG-BMI | TE | 0.13(0.018,0.238) | 0.024 |
| TyG-BMI | PM | 0.072(-0.004,0.323) | 0.062 |
| TyG-WC | NIE | 0.01(0.002,0.02) | 0.012 |
| TyG-WC | NDE | 0.159(0.05,0.258) | 0.002 |
| TyG-WC | TE | 0.169(0.061,0.269) | 0.002 |
| TyG-WC | PM | 0.061(0.009,0.199) | 0.014 |
| TyG-WHtR | NIE | 0.005(-0.003,0.014) | 0.250 |
| TyG-WHtR | NDE | 0.084(-0.028,0.179) | 0.116 |
| TyG-WHtR | TE | 0.09(-0.022,0.183) | 0.104 |
| TyG-WHtR | PM | 0.058(-0.26,0.402) | 0.318 |

Abbreviations: TyG: triglyceride-glucose; BMI: body mass index; WC: waist circumference; WHtR: waist-to-height ratio. CI, confidence interval; TE: total effect NDE: natural direct effect; NIE: natural indirect effect.

Model adjusted for sex, age, education, and marital status, residence, alcohol consumption, smoking, hypertension, diabetes, and dyslipidemia.

**Table S8 Sensitivity analyses of the mediating role of depressive symptoms in the relationship between obesity-related TyG index and mental status**

| **Exposure** | **Effect** | **Coefficient (95% *CI*)** | ***P*** |
| --- | --- | --- | --- |
| TyG-BMI | NIE | 0.005(0,0.009) | 0.038 |
| TyG-BMI | NDE | 0.071(0.023,0.118) | 0.004 |
| TyG-BMI | TE | 0.076(0.027,0.123) | 0.002 |
| TyG-BMI | PM | 0.062(0.004,0.163) | 0.040 |
| TyG-WC | NIE | 0.005(0.001,0.011) | 0.012 |
| TyG-WC | NDE | 0.094(0.047,0.141) | <0.001 |
| TyG-WC | TE | 0.1(0.052,0.147) | <0.001 |
| TyG-WC | PM | 0.052(0.008,0.133) | 0.012 |
| TyG-WHtR | NIE | 0.003(-0.002,0.007) | 0.250 |
| TyG-WHtR | NDE | 0.057(0.01,0.103) | 0.018 |
| TyG-WHtR | TE | 0.06(0.012,0.107) | 0.014 |
| TyG-WHtR | PM | 0.044(-0.044,0.215) | 0.256 |

Abbreviations: TyG: triglyceride-glucose; BMI: body mass index; WC: waist circumference; WHtR: waist-to-height ratio. CI, confidence interval; TE: total effect NDE: natural direct effect; NIE: natural indirect effect.

Model adjusted for sex, age, education, and marital status, residence, alcohol consumption, smoking, hypertension, diabetes, and dyslipidemia.
